# Genome Sequences of *Staphylococcus hominis* Strains ShAs1, ShAs2, and ShAs3, Isolated from the Asian Malaria Mosquito *Anopheles stephensi*

**DOI:** 10.1128/genomeA.00085-16

**Published:** 2016-03-10

**Authors:** Grant L. Hughes, Juan Antonio Raygoza Garay, Vikas Koundal, Jason L. Rasgon, Michael M. Mwangi

**Affiliations:** a Department of Pathology and Institute for Human Infections and Immunity, University of Texas Medical Branch, Galveston, Texas, USA; b Department of Biochemistry and Molecular Biology, Center for Infectious Disease Dynamics, Pennsylvania State University, University Park, Pennsylvania, USA; c Huck Institutes of the Life Sciences, Pennsylvania State University, University Park, Pennsylvania, USA; d Department of Entomology, Center for Infectious Disease Dynamics, Pennsylvania State University, University Park, Pennsylvania, USA

## Abstract

*Staphylococcus hominis* is a culturable component of the bacterial microbiome of *Anopheles stephensi*. Here, we present the annotated draft genome sequences of three *S. hominis* isolates from *A. stephensi*. These genomic resources will facilitate experiments to further our understanding of the role of bacteria in mosquito biology.

## GENOME ANNOUNCEMENT

Bacterial microbes profoundly influence many aspects of mosquito biology (1–3), and these traits have important ramifications for mosquito-borne disease (4). In addition to microbial interactions influencing vector competence, it is becoming evident that microbial interplay can also shape microbiome composition (5, 6). Here we isolated and sequenced three strains of *Staphylococcus hominis* from *Anopheles stephensi* laboratory-reared mosquitoes. This bacterial species is known to infect *A. stephensi* in the field (7).

A homogenate of surface-sterilized *A. stephensi* (Liston strain) was used to inoculate LB agar plates, and three colonies—ShAs1, ShAs2, and ShAs3—were isolated and confirmed by 16S rRNA gene sequencing to be *S. hominis*. Genomic DNA was extracted using a Qiagen Blood and Tissue kit following the recommendation for bacteria. The sequencing was done in a 500-cycle run on an Illumina MiSeq at the Pennsylvania State University Genomics Core Facility. All three DNA libraries were prepared using a Nextera XT DNA Library Preparation Kit and had an insert size of 400 bp. The 250-bp paired-end reads were initially assembled using MIRA version 4.0, and the assemblies were refined using DNAStar SeqMan Pro version 12.0. For ShAs1, this resulted in 17 contigs with a combined length of 2.1 Mbp, an *N*_50_ statistic of 332,788 bp, a median read coverage of 150×, and an average G+C content of 34%. For ShAs2, there are 16 contigs with a combined length of 2.1 Mbp, an *N*_50_ statistic of 393,276 bp, a median read coverage of 139×, and an average G+C content of 31%. For ShAs3, there are 15 contigs with a combined length of 2.0 Mbp, an *N*_50_ statistic of 288,000 bp, a median read coverage of 122×, and an average G+C content of 33%. The annotation was done using the RAST pipeline (8–10) followed by manual curation, yielding 2,021 protein-coding genes (CDSs) and 66 RNA genes for ShAs1; 2,044 CDSs and 46 RNA genes for ShAs2; and 1,889 CDSs and 59 RNA genes for ShAs3.

In the multialignment of the contig and read sequences for ShAs1, ShAs2, and ShAs3, polymorphisms could be called in 98% of the columns with false-positive and false-negative rates that are effectively zero. In these columns, no polymorphisms were detected between ShAs1, ShAs2, and ShAs3, indicating that the three colonies are nearly or exactly genetically identical. This suggests that the *S. hominis* population in the laboratory-reared *A. stephensi* has a high degree of genetic homogeneity. In the other 2% of the columns in the multialignment, polymorphisms could not be reliably called due to the ambiguous mapping of the reads due to the highly repetitive sequence.

*S. hominis* is a relatively common and harmless commensal of human and animal skin (11). Comparative genomics between microbes residing on humans and within mosquitoes may provide insights into the evolution of insect symbiosis. Furthermore, the draft genome sequences discussed here will add to the resources for the study of the *A. stephensi* microbiome—resources that include the sequences of *Stenotrophomonas* spp. (12) and *Elizabethkingia anophelis* (13) discussed elsewhere.

### Nucleotide sequence accession numbers.

This whole-genome shotgun project has been deposited at GenBank under the accession numbers LFKQ00000000, LFKR00000000, and LFKS00000000.
